# The systematic development of attributes and levels for a discrete choice experiment of HIV patient preferences for long-acting antiretroviral therapies in the United States

**DOI:** 10.1186/s12981-022-00435-6

**Published:** 2022-02-25

**Authors:** Aaron T. Brah, Douglas Barthold, Brett Hauber, Ann C. Collier, Rodney J. Y. Ho, Vincent C. Marconi, Jane M. Simoni, Susan M. Graham

**Affiliations:** 1 Division of Allergy & Infectious Diseases, Department of Medicine, University of Washington, Seattle, WA USA; 2 The Comparative Health Outcomes, Policy, and Economics (CHOICE) Institute, University of Washington, Seattle, WA USA; 3 Pfizer, Inc, New York, NY USA; 4 Department of Pharmaceutics and Bioengineering, University of Washington, Seattle, WA USA; 5 Division of Infectious Diseases, Department of Medicine, Emory University School of Medicine, Atlanta, GA USA; 6 Department of Global Health, Rollins School of Public Health, Emory University, Atlanta, GA USA; 7 Department of Psychology, University of Washington, Seattle, WA USA; 8 Department of Global Health, University of Washington, Seattle, WA USA; 9 Department of Epidemiology, University of Washington, Seattle, WA USA

## Abstract

**Introduction:**

Patient preferences for long-acting antiretroviral therapies (LA-ART) should inform development of regimens with optimal adherence and acceptability. We describe a systematic process used to identify attributes and levels for a discrete choice experiment (DCE) designed to elicit preferences for potential LA-ART options in the US.

**Methods:**

Our approach was conducted in four stages: data collection, data reduction, removing inappropriate attributes, and optimizing wording. We started with 8 attributes defining potential LA-ART products based on existing literature and knowledge of products in development. We conducted 12 key informant interviews with experts in HIV treatment. The list of attributes, the set of plausible levels for each attribute, and restrictions on combinations of attribute levels were updated iteratively.

**Results:**

Despite uncertainty about which products will become available, key informant discussions converged on 4 delivery modes (infusions and patches were not considered immediately feasible) and 6 additional attributes. Treatment effectiveness and frequency of clinical monitoring were dropped. Oral lead-in therapy was split into two attributes: pre-treatment time undetectable and pre-treatment negative reaction testing. We omitted product-specific systemic and local side effects. In addition to mode, the final set of attributes included: frequency of dosing; location of treatment; pain; pre-treatment time undetectable; pre-treatment negative reaction testing; and late-dose leeway.

**Conclusions:**

A systematic process successfully captured elements that are both feasible and relevant to evaluating the acceptability of potential LA-ART alternatives to patients.

## Key points

- Long-acting antiretroviral treatment regimens currently in development will increase choice and may improve clinical outcomes in persons with HIV infection.
- Creating a discrete choice experiment (DCE) based on hypothetical attributes of products in development requires detailed input from experts and stakeholders, but can provide important data to help guide product development and understand patient preferences.
- While key informant interviewers with product developers, researchers, and clinicians can provide guidance to inform DCE development, careful pilot testing will be needed to ensure the salience and comprehension of the different attributes and levels identified.

## Introduction

The development of long-acting antiretroviral therapy (LA-ART) is an important technological advance that could increase ART uptake and adherence by providing patients with new options to support viral suppression [1]. Historically, patients with HIV have had few alternatives other than oral pills. Early regimens that required multiple pills taken one or more times each day have become less complicated, with a clear advantage for single-tablet daily oral regimens in terms of adherence and viral load suppression [2]. Unfortunately, many patients still face challenges with ART initiation and adherence. Of the 1.1 million people living with HIV (PLWH) in the United States (US), approximately 86% have been diagnosed, 65% are receiving ART while in care, and 56% are virally suppressed (approximately 86% of those on treatment) [3]. The advent of new LA-ART modalities will provide increased options for patients who face barriers to taking daily oral pills. Indeed, an increasing body of evidence demonstrates patients are interested in and willing to try LA-ART [4–7].

Our own preliminary work showed that effectiveness and dosing frequency may be critical for LA-ART acceptability, and that patients vary in their preferences regarding side effects such as pain and injection-site reactions [8, 9]. Most LA-ART will have drawbacks that could reduce acceptability for some PLWH and even be “deal-breakers” for others, limiting uptake. Research is urgently needed to understand the LA-ART product attributes and individual patient characteristics that will drive end-user acceptability, so that developers can iteratively formulate more desirable products, funders can prioritize the products and delivery modes most acceptable to patients, and researchers can identify interventions and services that are most likely to result in high uptake and sustained use of LA-ART options.

In the present study, we conducted interviews with key informants (KI) knowledgeable about emerging technologies in the ART drug development pipeline to develop a set of attributes and levels for use in a discrete-choice experiment (DCE) designed to elicit preferences for a broad range of potential LA-ART products.

## Methods

### Development of preliminary attributes

**Fig. 1:**
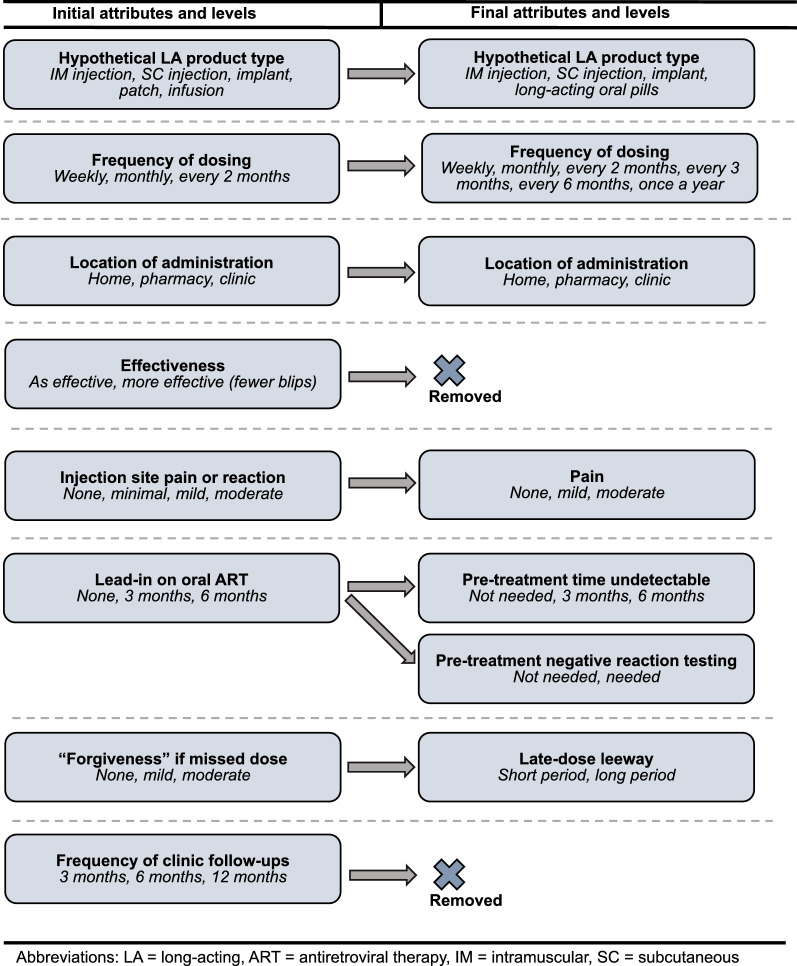
Initial and final attributes and levels. LA: long-acting; ART: antiretroviral therapy; IM: intramuscular; SC: subcutaneous

In developing attributes and levels, we followed a systematic process similar to Helter & Boehler’s four-stage model of attribute development: (1) raw data collection; (2) data reduction; (3) removing inappropriate attributes; and (4) wording [10]. First, we conducted a review of the relevant literature, including our previous research, to develop a preliminary set of attributes and levels defining potential new LA-ART products (Fig. 1) [6–9]. We then conducted 12 KI interviews with experts in HIV research, product development, and direct patient care who had knowledge of LA-ART products in development. The purpose of these interviews was to better understand the factors most likely to influence patient perceptions of the acceptability of different LA-ART regimens as alternatives to their current oral treatment regimen. Based on these interviews, we used an iterative approach to develop a final set of attributes and levels, including restrictions for combinations of attribute levels, for use in a future DCE.

### Development of topic guide for KI interviews

We developed a structured topic guide to be used in the KI interviews. The topic guide began with an introduction followed by four questions: (1) “Which LA-ART hypothetical products are most likely to come on the market in the next 5–10 years?” (2) “Which type of hypothetical product is most exciting to you and why?” (3) “What do you think would be the main competitor for this product, and why?” and (4) “What challenges do you foresee with providing combination LA-ART regimens to patients in the United States?” Additional probes included: “How do the challenges of providing LA-ART for HIV treatment differ from those of providing LA-ART for HIV prevention?” and “Do you think that challenges or preferences might differ for patients who are ART-naïve compared to those who are ART-experienced?”.

### KI interviews

The project leads (SMG, an HIV physician and clinical epidemiologist, and JMS, a clinical psychologist, each with content expertise) conducted the interviews using a HIPAA-compliant videoconference platform. A key assumption that was made explicit to KIs at the beginning of each interview was that the efficacy of all hypothetical products developed would be equivalent, and that none of these products would be considered an HIV cure. This assumption allowed us to focus specifically on which attributes and levels most clearly differentiate hypothetical products.

### Iterative development of attributes and levels

After responding to the questions in our topic guide, each KI was presented with the most up-to-date table of attributes and levels under consideration and then asked to provide feedback. Specifically, KIs were asked for their opinions on attribute descriptions, attribute levels, and restrictions on combinations of attribute levels for different delivery modes such as injections or implants. Each KI was also asked to comment on the scope and direction of the study specifically and on possible patient preferences for LA-ART in general.

The table was updated iteratively throughout the KI interviewing process, with decisions to modify the table based on input received during each KI interview and weighed relative to the cumulative responses from all previous KI interviews. Immediately after each interview, brief summary notes [11] were circulated to study team members for discussion at a weekly team meeting. Language complexity and wording were carefully considered and edited to make the information more comprehensible for the patient population.

After all KI interviews were completed and consensus among the study team members was achieved, the final attribute and level table, including restrictions on combinations of attribute levels was sent to all KIs for final feedback. Examples of feedback from the KIs included comments indicating that feasible frequencies of treatment for LA oral medications range up to 4 weeks, and that intramuscular (IM) injections could not realistically be administered at home.

### Transcription and qualitative coding of KI interviews

Interviews ranged from 45 to 60 min. Audio recordings of the interviews were processed through an audio-to-text transcription program, then reviewed and edited for accuracy by two team members (ATB, SMG). Final edited transcripts were circulated to the study team members for further review and discussion.

After completion of all KI interviews, we began a more detailed analysis of the transcripts via thematic coding focused on KI quotes that led to modification or refinement of the DCE attributes and levels. A codebook was created with codes that referenced the specific topic guide questions used in each interview. These codes were then broken down into sub-codes specifying the mode of medication delivery discussed. After the codebook was completed, each interview transcript was uploaded to the qualitative analysis software (Dedoose, Hermosa Beach, CA, USA) for coding by ATB, with review of coded transcripts by JMS or SMG for consistency.

During the qualitative analysis process, two new codes were added to the codebook to capture important information that was not specifically elicited in the KI interview topic guide questions: (1) LA-ART products least likely to reach the market soon and (2) suggested changes to wording or presentation of the attributes and levels. For the themes related to wording and presentation, we also coded whether the study team rejected or accepted each KI-suggested change. We used Dedoose to identify specific quotes that highlighted the most salient themes.

## Results

### Participant characteristics

The 12 participants comprised 5 women and 7 men, with ages ranging from 38 to 66 years. KIs main expertise was in clinical research (9), clinical practice (1), sociobehavioral research (1), and pharmaceutical research (1). The median years of experience working in the HIV field was 23 years (range = 3–38 years).

### Themes from KI interviews

The major themes that emerged from KI interviews involved which products were most likely to be developed, should be removed from consideration or had significant barriers to development or roll-out, and which attributes were likely to be considered most desirable by patients. All KIs agreed with our assumption that all products considered would be effective at suppressing viral load but not lead to a cure.

### Identification of products most likely to be developed

Among the original treatment modes considered, IM injections, subcutaneous (SC) injections, and implants were retained as the emerging technologies most likely to become available for PLWH. LA oral tablets were added as a promising option, because KIs thought these would likely be available in the next 5–10 years. Among these four feasible options, most KIs (9 total) predicted that IM regimens were most likely to be available in the near future:

*“Obviously, the cabotegravir/rilpivirine [regimen] is on the way…I'm pretty confident that that'll get moving forward. And I'm thinking we'll see some of those formulations get put together in the future in ways that allow less frequent dosing.”* – KI#5

SC injection-based regimens were also frequently referenced (5 KIs) as a feasible next-in-line option:

*“I think there will be high interest in injectables given in clinics, which still could be either intramuscular or subcutaneous….And I think…to be explicit about subcutaneous…with the right formulation, a subcutaneous injection could be given at home.”* – KI#9

Apart from injectable products, most KIs (9 total) considered implants to be promising:

*“I'm most excited about the [Islatravir] implant… The pharmacokinetic properties are amazing. And I think… there's human data and it looks good. So, I'm very optimistic about that drug.”* – KI#10

Finally, LA oral tablets were mentioned by two of our experts as a potentially exciting option for patients:

*“I think that they [LA oral tablets] would be an option...We have the paradigm of long-acting tablets being acceptable for other conditions like osteoporosis. So I think that…would be a great option for certain patients. In some ways, a long-acting oral tablet…would be a lot more acceptable to patients for whom the IM injections are a challenge.”* – KI#1

### Removal of less promising products and certain attributes

Both infusions and patches were included in the original list of possibilities, but were dropped from consideration based on KI input. Regarding infusions:

*“I think it’s going to be so difficult and probably so expensive… it might be interesting from a scientific point of view, potentially in cure research. But in terms of widespread rollout of infusions for treatment of the millions of people in the world that need it…I don't see that that's a realistic probability.”* – KI#2

Regarding our query about whether to keep patches in the list of options, a pharmaceutical researcher stated:

*“When we talk about the patch, we talk about the Durogesic transdermal patch and other analgesics...these are highly potent drugs where you need a very low dose… and are only used for once-a-day or more frequent administrations… But for HIV drugs, as you know….none of the drugs are as potent.”* – KI#12

Because there were several different modes to evaluate, we opted to drop injection site reaction as a component of the pain attribute. Indeed, when we debated the inclusion of an attribute on side effects, it became clear that this would complicate the choice tasks, since different pharmaceutical agents with the same mode of delivery could have very different side effects. One clinical researcher confirmed this challenge:

*“I think you're right, because there's so many different side effects.. and from a patient's point of view or physician’s point of view,… the ones that are irreversible … that lead to end-organ damage … are the ones that people are more likely to be concerned about. Or, in reality, the ones that accumulate over 10 or 20 years… since we're talking about a timeframe of…lifelong therapy.”* – KI#9

Finally, because most clinician KIs thought that the schedule for clinic visits and laboratory monitoring would not differ no matter how treatment was delivered, we omitted an attribute on the frequency of clinic visits for medical review.

### Product attributes likely to influence acceptability

KI discussions confirmed several product attributes on our initial list (Fig. 1) that they felt would influence acceptability. These included less frequent dosing, treatment administration at a convenient location, minimal injection or insertion site pain (for injections or implants), and a reasonable leeway (i.e., window) for safe re-dosing in the event of a missed or delayed administration. When asked how best to present a late-dose leeway attribute, most KIs thought it would be reasonable to make the leeway interval proposed correspond to a percentage of a given hypothetical product’s dosing interval. For example, one clinical researcher declared:

*“I'd be surprised if you even needed 25%...I mean it might make a difference, for example, for some of the oral long-acting…the shorter intervals of 25% or 50%. But for the injectables, I'd be surprised if you don't have a plus or minus 2-week tail, something like that….Anyway, I think 25%, 50% and 100% is fine.”* – KI#5

Most KI felt it is important to include information on pre-treatment lead-in to monitor for adverse events or ensure viral suppression before switching to a LA-ART regimen, since these requirements were included in the clinical trials leading to approval of LA cabotegravir/rilpivirine:

*“…given the data, cabotegravir and rilpivirine are probably going to come out [in injectable form]… I mean, it's hard for me to believe that the FDA is going to not have them mimic what they've done in their trials, which was 6 months [oral lead-in time].”* – KI#9

Especially with respect to adverse event monitoring, KIs felt that HIV care providers would emphasize this requirement:

*“…Some docs absolutely want the oral lead-in because they want the reassurance that there's not going to be an allergic reaction…, because of course once you have given the shot, it hangs around for as much as a year. So, if you have a hypersensitivity reaction that would be a worry.”* – KI#6

Finally, when asked about possible levels for the oral lead-in attribute, most KIs agreed it would be best to separate the attributes for adverse event monitoring vs. viral suppression. For example:

*“..with regards to the lead-in… I think that's good to separate that from allergy to the need for viral suppression, because I think those are two completely separate questions. And especially if eventually the medication that's used for viral suppression ends up being the one that's also injected too. So that might change the equation a little bit.”* – KI#1

### Additional feedback about products and attributes

The most salient concerns KIs expressed about the use of LA regimens included a preference for not switching from a successful daily oral regimen, logistical barriers to the clinical delivery of injections and implants, and the potential for stigma due to visible scarring or stigmata of use (e.g., an implant visible under skin). Many KIs suggested a moderate likelihood that patients will choose to stay with their daily oral tablets when presented with these LA regimens. For example, a clinician stated:

*“You know, I think some individuals may prefer the routine of daily dosing. I have a lot of folks who have been on a regimen for years that are very hesitant to change and resist suggestion for change.”* – KI#8

For this reason, we included a constant comparison to the patient’s current daily oral regimen in the DCE design.

Another concern expressed by many of the KIs when discussing the forthcoming regimen delivered by IM injection every 1–2 months was that it would place a logistical burden on the clinics where patients would come to receive their scheduled dosing. According to one clinical researcher:

*“…The logistics are something we're already worrying about here. Because people that are doing well on their therapy will come in every 6 months. We have some even just come annually and if they have to come to the clinic every month, just in terms of the sheer volume of people to accommodate and finding rooms to do the injections and the staff to do it…”* – KI#2

Finally, one KI presented us with the consideration that LA regimens will likely require two or three different drugs effective against the patient’s HIV strains, resulting in at least two drugs to be administered:

*“One thing that may be important that I don't see here, is a trade-off between duration and number. The number of injections versus the duration or the number of implants versus duration and/or tablets… because, and again, in prevention, what we've seen is that…people prefer a longer duration, even if they need more than one injection or more than one rod… So I think that's a really important one to think through, given the products you are considering…and I am almost going to tell you that you know for an implant, there is very little chance that you could have a tri-therapy in a single implant. Most likely, you will have to have two rods.”* – KI#7

### Final attribute list

Completion of KI interviews with iterative updating led to a final set of attributes and levels with restrictions as detailed in Table 1, reflecting the following changes:

1. Dropping microneedle patches and infusions as product types and adding LA oral tablets as an option;
2. Including frequency options of 3 months, 6 months, and 1 year for product types expected to have a long duration of potency, such as implants;
3. Removing effectiveness as an attribute since viral suppression would be required for product approval;
4. Removing injection site reactions as a component of the injection site pain attributes and removing consideration of any drug-specific side effects, as these are likely to vary across product types and are unknown for many products;
5. Modifying the levels for injection site pain to “none”, “mild”, or “moderate” and eliminating “minimal” as it is hard to distinguish from “mild;”
6. Splitting the lead-in on oral ART into two attributes: one for pre-treatment viral suppression (labelled “pre-treatment time undetectable”) and the other for pre-treatment adverse event monitoring (labelled “pre-treatment negative reaction testing”);
7. Simplifying the levels for pre-treatment time undetectable to “not needed,” 3 months, or 6 months prior to the switch to LA ART;
8. Simplifying the levels for negative reaction testing to “needed” versus “not needed;”
9. Simplifying the levels for late-dose leeway to shorter or longer periods based on the frequency of dosing (i.e., 25%, 50% or 100% of the dosing interval for that option in the choice set); and
10. Dropping the attribute for frequency of clinical follow-up, as previously discussed.

**Table 1:** Feasible restrictions placed on attributes and levels for each mode of treatment

Attribute	Levels	Restrictions by mode of administration				
		LA Oral	SC injection	IM injection	Implant	All modes
Mode	LA Oral, SC Injection, IM Injection, Implant*					
Frequency	1, 4, 8, 12, 26, 52 weeks	1 or 4 weeks	1, 4, 8, or 12 weeks	4, 8, or 12 weeks	26 or 52 weeks	IF location = clinic or pharmacy, THEN dose frequency ≥ 4
Location	Home, pharmacy, clinic	Only home	Any	Not home	Only clinic	IF frequency = 1 week, THEN location = home
Pain	None, mild, moderate	Only none	None or mild	Mild or moderate	Mild or moderate	
Oral lead-in	0, 3, 6 months					
Negative reaction testing	Yes or no					
Late dose leeway	Short, long*					

^*^Long-acting oral tablet, subcutaneous injection, intramuscular injection

^*^Short vs Long: For the late dose leeway attribute, the duration of time displayed to respondents for both the "short" and "long" levels was dependent on the dosing frequency of that alternative

In addition to these changes in attributes and levels, which are summarized in Fig. 1, we decided that the final DCE design will include a constant comparison to the patient’s current daily oral regimen and that our descriptions of each product would include two administrations by mode (i.e., two injections, two implants), given the need for multidrug therapy.

## Discussion

We used an iterative developmental process over the course of 12 key informant interviews coupled with qualitative content analysis of interview transcripts, to develop a final list of attributes and levels to be used in an upcoming DCE to elicit the impact of different treatment features on the likely acceptability of LA ART. After each interview, the research team agreed upon the most salient KI suggestions and used these to inform possible additions, deletions, or modifications to any of the pre-specified attributes and levels. The table of attributes and levels was modified each week based on KI suggestions before subsequent interviews were performed. This ensured each KI had the opportunity to provide commentary on the attributes and levels deemed most feasible up to that point. Our final results include specific attributes and restricted levels related to IM injections, SC injections, implants, and LA oral regimens: the four modes of delivery considered most likely in the near future.

Recent work on specific HIV prevention and HIV treatment regimens in development or recently approved for use has highlighted the importance of considering patient preferences regarding delivery modes, dosing frequency, delivery location, and injection-site pain [12–14]. That said, there has not yet been an attempt to address the full spectrum of potential LA-ART product modes or to evaluate the impact of factors such as pre-treatment time undetectable, pre-treatment negative reaction testing, and late-dose leeway on patient preferences. The current work provides a basis for research on patient preferences for a large range of hypothetical LA-ART products, with attributes and levels derived from detailed discussions with key informants.

While considerable research on patient preferences for LA treatments exists in the areas of HIV prevention, hormonal contraception, and schizophrenia treatment [12, 15–18] none of the existing literature on patient preferences regarding specific LA-ART regimens explicitly asks PLWH to contextualize their preferences in contrast to their current daily oral ART regimens. Our decision to develop a more comprehensive list of LA-ART products, attributes, and levels, with a constant comparison to patients’ current daily oral ART reflects what most patients would experience in actual practice, should new LA-ART products become available to them. This direct comparison could provide important insight into not only which future LA-ART regimens are most preferable generally, but also which are more attractive to specific sub-groups of PLWH. For example, we postulate that patients who take multiple daily oral pills in addition to their ART will be more likely to prefer maintaining their daily oral ART regimen as they would not likely perceive the addition of just one or two more medications to be troublesome.

One of the primary strengths of this research is that we spoke directly with a variety of experts who are knowledgeable about emerging HIV treatment technologies and HIV patient care. This is important because we are primarily interested in assessing patient acceptability of products that are likely to come to market—rather than assessing preferences for products ideal for the patient, but not currently feasible. In this way, we can preemptively elicit realistic and meaningful patient preferences for technologies that are likely to actually appear on the market and become an alternative to their current daily oral ART. Another strength of this work is that we have been able to explicitly develop procedures to elicit patient preferences regarding the need for lead-in oral therapy to monitor for adverse events or to ensure viral suppression prior to a switch to LA-ART, and the leeway for redosing if a dose is missed. These factors are likely to become salient when new technologies become available and patients and their clinicians discuss the process of switching from their current daily oral ART to a LA-ART regimen. Such data will be valuable as drug developers and service planners consider the likely logistical and technical challenges of LA-ART product roll-outs.

A limitation of this work is that we did not specifically include patient feedback in this early phase of DCE development. While we believe that the input from the KIs on patient perspectives is likely to be accurate, pilot testing and direct feedback from patients on our final attributes and levels, and their wording, will be critical and is planned. Another challenge we encountered involves the correlation of many of the attributes. For example, dosing frequency will likely always be correlated with mode of administration. This correlation between attributes led to the requirement for a number of restrictions on the combinations of attribute levels. These restrictions need to be taken into account in analyzing our DCE data, and their impact on modeling our results will require careful checking during our pilot testing phase.

A final possible barrier inherent in this work is that we chose not to consider costs, product-specific side effects, or logistical constraints related to the roll-out of specific LA-ART products. In order to develop a design that could extract raw patient preferences, we intentionally left out these factors and kept to hypothetical choice sets. While we believe that products matching our hypothetical choice set combinations are technologically feasible, there is a possibility that cost, toxicity, or logistical factors could eventually render them unrealistic.

## Conclusion

In conclusion, as new LA-ART products come to market, the range of treatment options will increase for patients with HIV and their providers. Novel options may improve clinical outcomes by promoting adherence to therapy, which remains a challenge for many patients. Identifying patient preferences, as well as patient characteristics associated with preferences for different treatment modes and attributes, may prove valuable for product developers, clinical researchers, and health systems as they develop, test, and disseminate new regimen options for patients needing lifelong HIV treatment. Ideally, LA-ART options will enhance not only adherence, which is critical to HIV outcomes, but also quality of life.

## Data Availability

The datasets used and/or analysed during the current study are available from the corresponding author on reasonable request.
